# Bilateral duplex collecting system with bilateral vesicoureteral reflux: a case report

**DOI:** 10.1186/s13256-019-2058-z

**Published:** 2019-05-04

**Authors:** Diataga Sylvestre Yonli, Marouene Chakroun, Selim Zaghbib, Delphine Ye, Abderrazak Bouzouita, Amine Derouiche, Mohamed Riadh Ben Slama, Haroun Ayed, Mohamed Cherif, Mohamed Chebil

**Affiliations:** 1 Department of Urology, Yalgado Ouedraogo University Hospital of Ouagadougou, Ouagadougou 03, 03 BP 7022 Burkina Faso; 2 Department of Urology, Charles Nicolle University Hospital of Tunis, Boulevard du 9 Avril 1938, 1006 Tunis, Tunisia

**Keywords:** Bilateral, Duplex collecting system, Vesicoureteric reflux, Heminephrectomy

## Abstract

**Background:**

A bilateral duplex collecting system is an unusual renal tract abnormality. Vesicoureteral reflux may be associated. We describe a rare case of bilateral duplex collecting system with bilateral vesicoureteral reflux in which the refluxing ureter on the left side drains the upper pole moiety contrary to what is often found.

**Case presentation:**

A 24-year-old married Arab woman presented with ascending left-sided flank pain during micturition. She complained of recurrent urinary tract infections. A physical examination and laboratory tests were normal. Voiding cystourethrography and computed tomography scan detected bilateral duplex collecting system, grade IV vesicoureteral reflux on the left side, and grade I vesicoureteral reflux on the right. She underwent left heminephrectomy and dextranomer/hyaluronic acid injections on the right side. After a year of follow-up, a clinical examination and imaging findings were unremarkable.

**Conclusions:**

A bilateral duplex collecting system with refluxing upper pole moiety ureter is a very rare entity. The diagnosis should be suspected when exploring any flank pain with recurrent urinary tract infections to avoid subsequent renal impairment. Furthermore, this case shows how some common symptoms may lead to finding an unexpected urinary tract abnormality.

## Introduction

A bilateral duplex collecting system is an unusual renal tract abnormality. Duplication occurs when two separate ureteric buds arise from a single Wolffian duct [1]. Based on the degree of fusion, it can present as bifid renal pelvis, partial ureteric duplication, incomplete ureteric duplication with ureters joining near or in bladder wall, and complete ureteric duplication with separate ureteric orifices [1]. According to the Weigert–Meyer law, the upper pole ureter typically opens medially while the lower pole ureter opens laterally [2]. The incidence of ureteral duplication has been reported as 1 in 125 or 0.8% [3].

The case that is presented does not seem to fit this law. Although bilateral vesicoureteral reflux (VUR) and bilateral duplex collecting system may be seen, their association with refluxing upper pole ureter is a rarely encountered entity. The aim of this observation is to present a rare case and its therapeutic aspects in a young married woman.

## Case presentation

A 24-year-old married Arab woman had been admitted to a local health center 2 months prior to referral to our urology department. She had been hospitalized there four times in 1 year for acute pyelonephritis. The fourth episode raised the suspicion for an underlying problem and justified her referral to our urology department after management of the acute pyelonephritis.

On admission, she complained of ascending left-sided flank pain during micturition but did not have dysuria or hematuria. She also had a history of frequent urinary tract infections (UTIs) as a young adult.

She was perfectly asymptomatic on the right side. A physical examination was normal. Her temperature was 37.4 °C, her blood pressure was 128/84 mmHg, and her pulse rate was regular at 76 beats per minute. Laboratory tests were normal; in particular, a urine examination showed no leukocyturia or bacteriuria.

She underwent an abdominal ultrasound which showed an asymmetric size of the kidneys and a bilateral chronic pyelonephritis aspect. Her right kidney measured 10 cm while the left measured 12 cm.

**Fig. 1:**
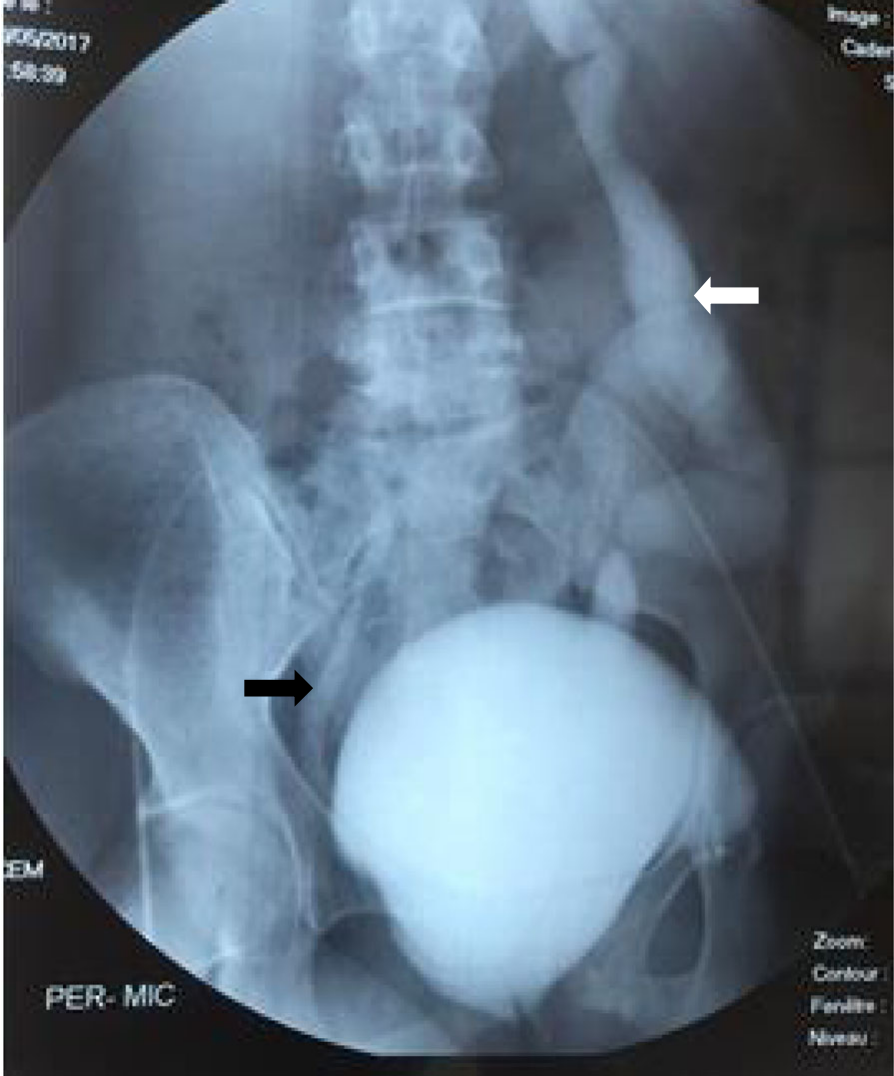
Voiding cystourethrography imaging showing bilateral reflux grade IV vesicoureteral reflux on the left (*white arrow*) and grade I vesicoureteral reflux on the right (*black arrow*)

**Fig. 2:**
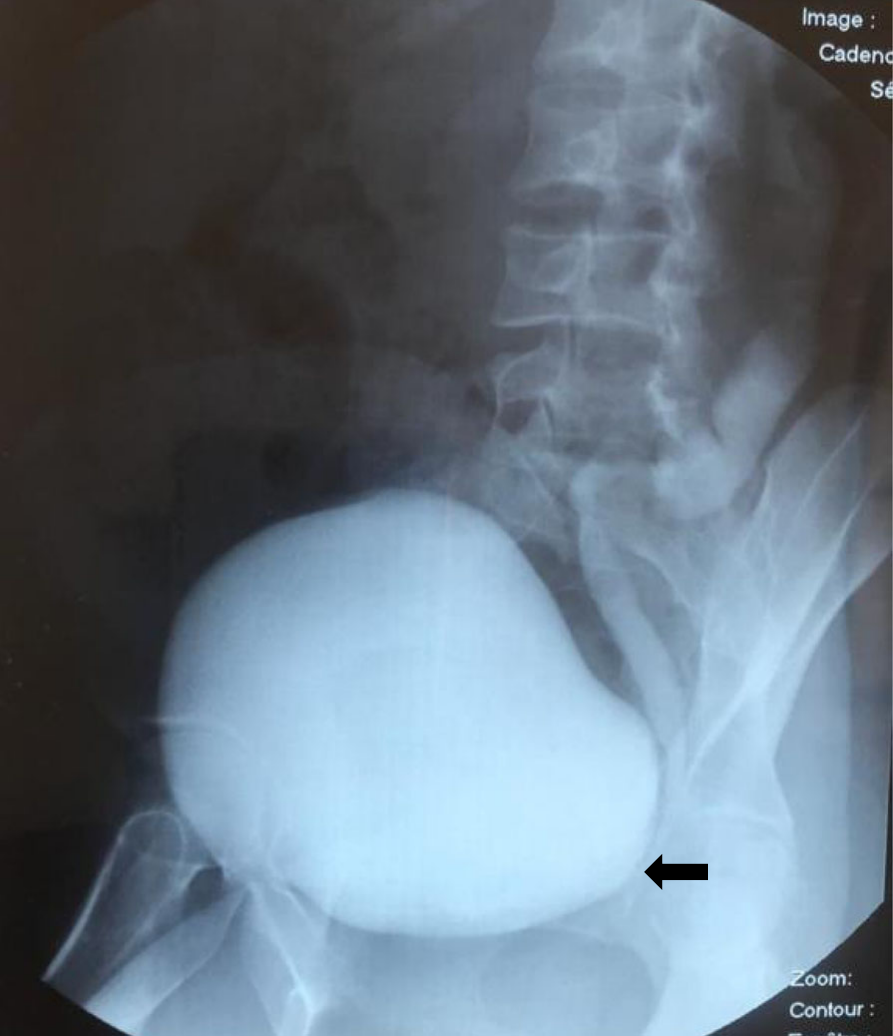
Voiding cystourethrography imaging showing left grade IV VUR (*arrow*)

A voiding cystourethrography (VCUG) was performed and showed grade IV VUR on the left side and grade I VUR on the right (Figs. 1 and 2).

**Fig. 3:**
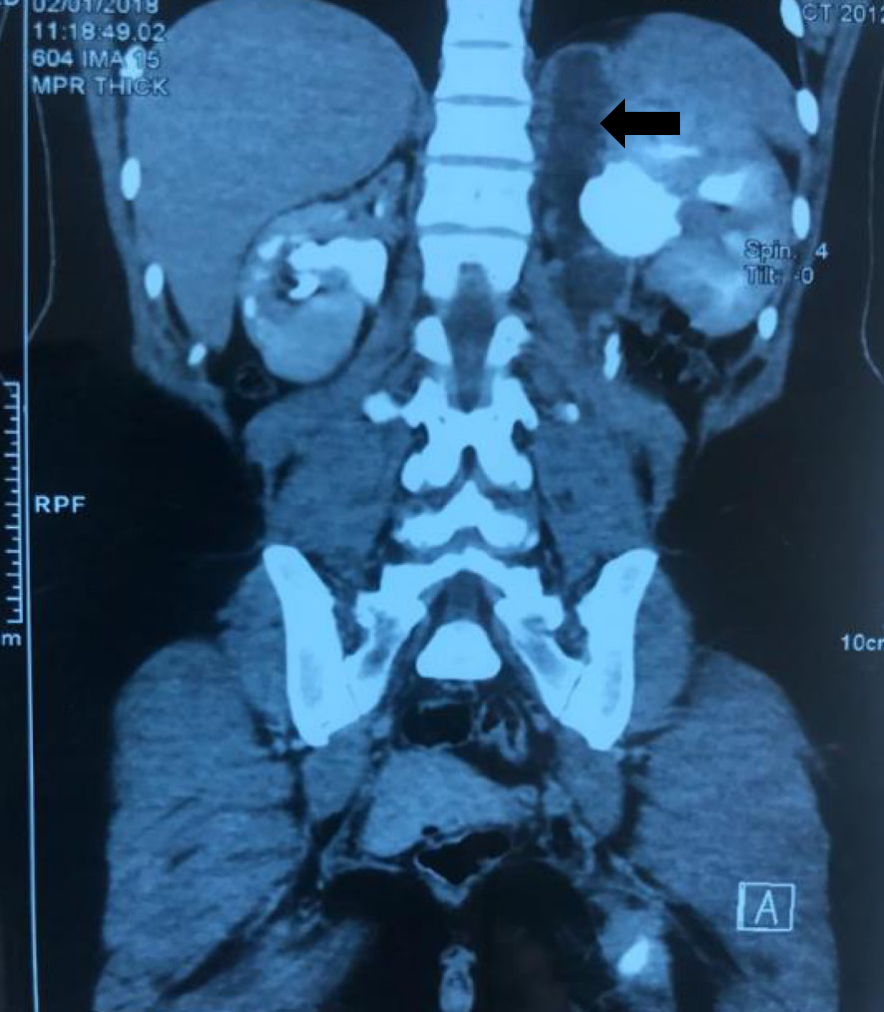
Coronal view of computed tomography scan with poor opacification of left upper pole moiety (*arrow*)

**Fig. 4:**
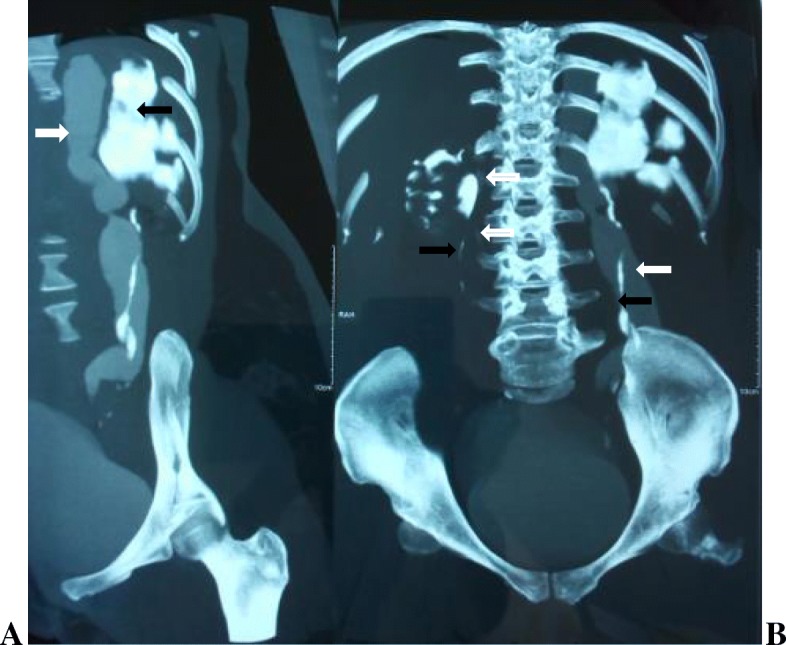
Excretory phase of computed tomography scan. **a** A 3/4 left view showing the upper pole moiety (*white arrow*) and the lower pole moiety (*black arrow*). **b** Bilateral duplex collecting system with hydroureteronephrosis of the left upper collecting system. The upper pole moieties and their ureters are poorly opacified (*white arrows*) especially on the right. The *black arrows* show the lower pole moiety ureters

An abdominal and pelvic computed tomography (CT) scan detected a left completely duplicated collecting system with hydroureteronephrosis and poor opacification of the upper pole moiety. In addition, the parenchyma of the upper pole moiety was atrophied with secretory and excretory delay. In association with VCUG findings, it appeared that the refluxing ureter was the one that drains the upper pole moiety and inserts lower into the bladder. On the right, a duplex collecting system was detected with hypotonic calyces, pelvis, and ureter of the upper pole moiety. An atrophic parenchyma and poor opacification of the upper pole moiety was also detected (Figs. 3 and 4). Renal scintigraphy was not available.

**Fig. 5:**
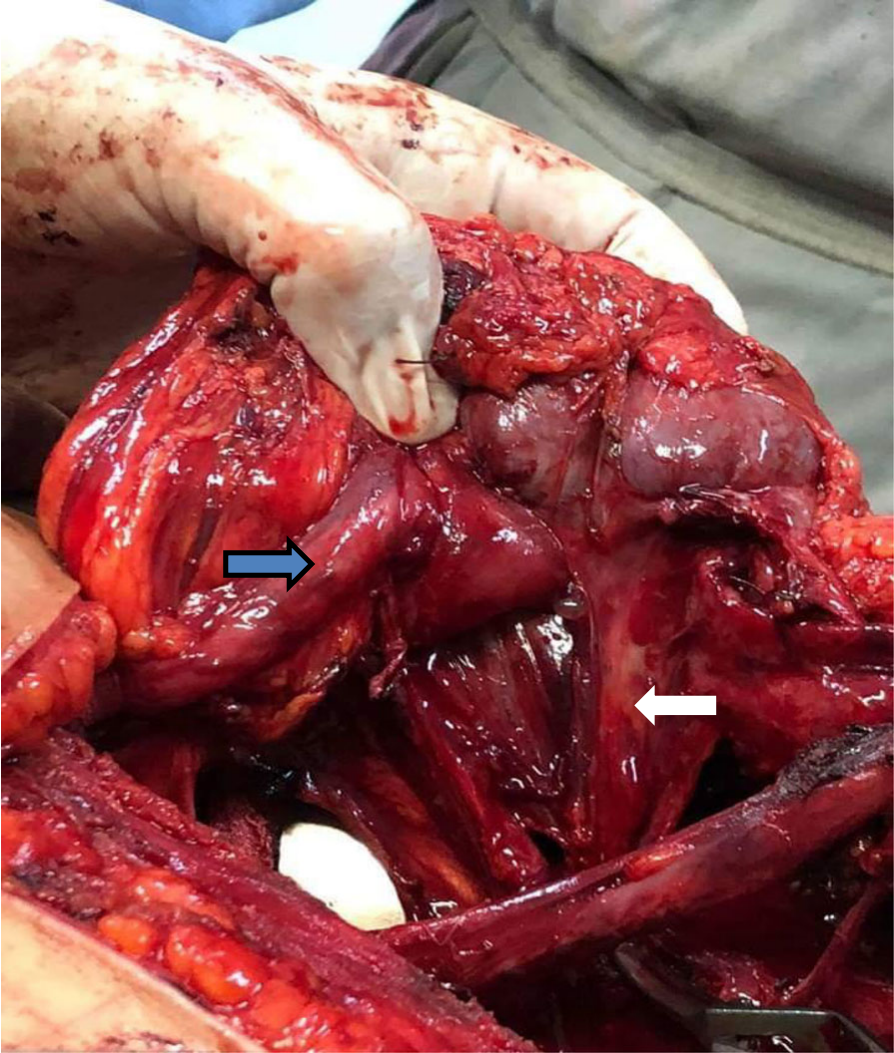
Intraoperative picture during left heminephrectomy showing left duplicated collecting system with the upper pole ureter (*white arrow*) and the lower pole ureter (*blue arrow*)

We carried out a left heminephrectomy because of the poor functioning of the upper pole moiety based on imaging findings associated with recurrent UTIs (Fig. 5). On the right side she underwent dextranomer/hyaluronic acid (Deflux®) injections. Dextranomer/hyaluronic acid (Deflux®) was injected submucosally below the ureteral orifice at the 6 o’clock position to create a prominent bulge and raise the distal ureter and ureteral orifice.

**Fig. 6:**
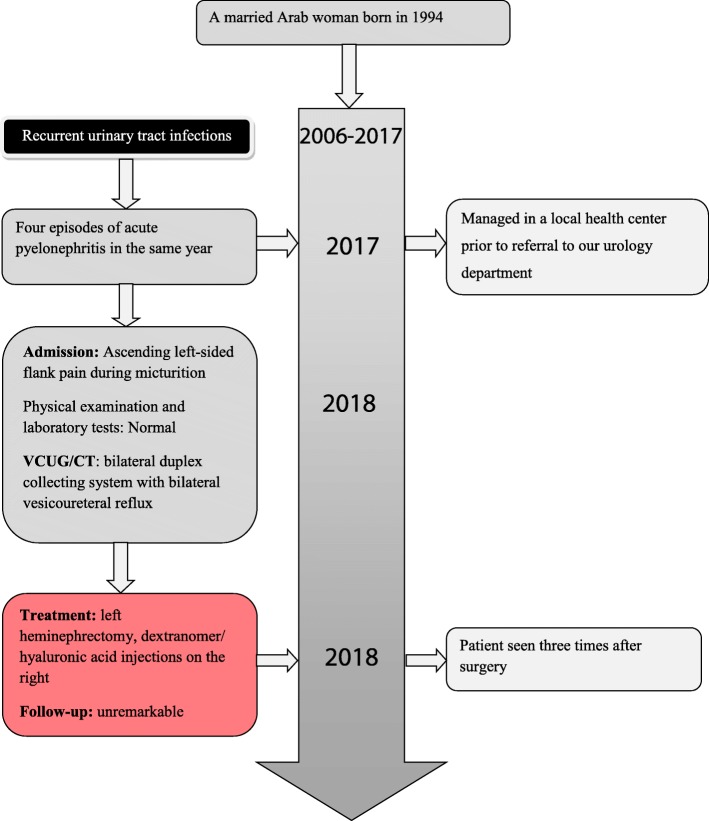
Timeline of the case. *CT* computed tomography, *VCUG* voiding cystourethrography

A year after the surgery she has no complaints. The symptoms are completely resolved. Biological and radiological follow-up is unremarkable. A timeline of the case is presented in Figure 6.

## Discussion

In this case we present a bilateral duplex collecting system and bilateral VUR. The limitation of our approach is the duration of the follow-up which is only approximately a year. The discussion has two sections: first, we will point out the originality of the abnormality; second, we will talk about the therapeutic aspects.

### An exceptional abnormality

Bilateral duplex collecting system is a rare abnormality. It occurs in 1 in 500 persons and is found in 0.3% of excretory urograms [3]. Some cases have been reported on bilateral duplex collecting system [4, 5]. There are not much data about it. The cases reported in the literature are mostly related to unilateral duplex collecting system.

In keeping with the Weigert–Meyer law, the upper pole ureter typically opens medially while the lower pole ureter opens laterally. Complete ureteral duplication may be associated with other congenital anomalies such as a short lower moiety intramural ureter causing VUR or an upper moiety ureter with an ureterocele causing obstruction [3, 6].

VUR almost always occurs into the lower pole moiety due to its lateral displacement within the bladder [7]. In the case that is presented, the refluxing ureter on the left side is the one that inserts lower into the bladder. Furthermore, there is no ureterocele. These situations are not common. Some rare cases of refluxing upper pole moiety have been mentioned in the literature [6]. However, the association of bilateral duplex collecting system, bilateral VUR, and refluxing upper pole moiety is not clearly described in the literature to the best of our knowledge.

### Therapeutic challenge

We had a case of bilateral VUR and recurrent UTIs associated with renal parenchyma destruction of left upper pole moiety in a 24-year-old married woman. She may develop these UTIs during pregnancy. Acute pyelonephritis during pregnancy significantly increases the risk of anemia, septicemia, acute renal failure, respiratory distress, spontaneous preterm birth, and low birthweight birth [8]. The main goal then was to protect our patient from these eventual consequences and most importantly to prevent renal failure [9]. On the left upper pole moiety a heminephrectomy was performed. In most cases this surgery contributes on the one hand to stopping the symptoms and on the other hand to avoiding kidney damage. An upper pole heminephrectomy is best applied to patients with non-functioning upper pole [4].

On the right side we had a grade I VUR on the upper pole moiety ureter. This side was perfectly asymptomatic. The majority of lower grades of VUR spontaneously resolve as the child grows [2]. This woman was 24-years old. We did not expect a spontaneous regression in this patient. To correct the reflux, an endoscopic injection of dextranomer/hyaluronic acid (Deflux®) was performed.

Several bulking agents have been used over the past two decades in the treatment of VUR. In 2001, the US Food and Drug Administration (FDA) approved the use of dextranomer/hyaluronic acid as the first FDA-approved subepithelial injectable treatment for VUR in the USA [10].

Finally, we may note that a bilateral duplex collecting system with refluxing upper pole ureter is rare. Furthermore, its association with recurrent UTIs in a young woman remains a therapeutic challenge.

## Conclusion

A bilateral duplex collecting system is a rare anatomic abnormality. Furthermore, the fact that the left refluxing ureter drains the upper moiety pole makes this case an exceptional case. The recurrent UTIs that are associated may cause renal damage. The therapeutic aspects of this are very important because our patient is a young, married, and procreative woman. A diagnosis and early treatment before any renal impairment seem to be the best approach.
